# Regenerative potential of partially differentiated mesenchymal stromal cells in a mouse model of a full-thickness skin wound

**DOI:** 10.17179/excli2018-1504

**Published:** 2018-08-31

**Authors:** Ausra Liubaviciute, Vytautas Kaseta, Aida Vaitkuviene, Zygmunt Mackiewicz, Gene Biziuleviciene

**Affiliations:** 1 State Research Institute Centre for Innovative Medicine, Department of Stem Cell Biology, Santariskiu str. 5, LT-08406 Vilnius, Lithuania; 2 State Research Institute Centre for Innovative Medicine, Department of Regenerative Medicine, Santariskiu str. 5, LT-08406 Vilnius, Lithuania

**Keywords:** mesenchymal stromal cells, partial differentiation, keratinocyte-conditioned medium, skin regeneration

## Abstract

Mesenchymal stromal cells (MSCs, known as mesenchymal stem cells) are considered to be a promising therapeutic tool for many diseases. But it is still unclear which cells are more efficient and safe for wound healing and tissue regeneration for clinical applications: undifferentiated, partially differentiated stem cells or differentiated cells. In this study, we modified MSCs with keratinocyte-conditioned medium (KCM) and examined MSCs, partially differentiated MSCs (PMSCs) and differentiated cell migration, accumulation in the wounded area as well as cell regenerative efficiency in a full-thickness skin wound model. In addition to that, the impact of intradermal and intravenous cell delivery methods of wound healing was evaluated. C57BL/6J mouse compact bone MSCs were treated with a KCM for 14 days. Flow cytometry analysis showed the appearance of keratinocyte surface markers which were absent in MSCs, whereas the specific markers for MSCs were lost. Cells were injected either intravenously or intradermally in C57BL/6J mice. Wound closure, cell migration and accumulation in the wounded area were further analysed. Wound healing was assessed by the rate of wound closure and by histological evaluation. Cells were monitored using optical imaging. We demonstrated that PMSCs showed morphology similar to keratinocyte cells, had enhanced migration and increased survival at the site of injury. PMSCs had a beneficial effect on wound healing and tissue regeneration. This effect was reinforced when these cells were injected intravenously. Due to their partial differentiation status, we assume that PMSCs can differentiate more rapidly into epidermal cell lineages thus causing faster and qualitatively improved wound healing.

## Introduction

Despite the considerable progress in the wound care, certain wounds heal improperly and become chronic. These wounds are associated with a significant patient morbidity and mortality (Frykberg and Banks, 2015[10]; Otero-Viñas and Falanga, 2016[22]). Present treatment options for chronic wounds (including gene therapy, cellular factors, engineered skin or skin equivalents) are limited and not always effective (Al-Shaibani et al., 2016[2]). 

Cytotherapy for the cutaneous wounds has recently come under investigation as a potential treatment modality for impaired wound healing. Among various types of cells, mesenchymal stromal cells (MSCs, known as mesenchymal stem cells) are considered to be one of the possible candidates for cell-based therapy. It is shown that during wound healing, MSCs enhance angiogenesis, accelerate reepithelialization and granulation tissue formation, modulate inflammation and regulate extracellular matrix (Lee et al., 2016[14]). How precisely stem cells contribute to the skin wound healing remains debated. Several assumptions were proposed, including direct MSC differentiation (e.g. into skin-like cells) (Chen et al., 2012[4]; Chavez-Munoz et al., 2013[3]) and indirect MSC action such as secretion of factors that affect fibroblast or keratinocytes migration, proliferation into the injury site and metabolic (Chen et al., 2008[5]; Ennis et al., 2013[9]; Dong et al., 2017[7]).

According to numerous research reports, MSC therapy is considered to be safe and well-tolerated (Zhao et al., 2016[38]; Daltro et al., 2017[6]). However, there are also reported investigations without observed benefits or even with the adverse effect under certain conditions (Rustad and Gurtner, 2012[26]; Frykberg and Banks, 2015[10]). 

It is considered, that the possible negative effect of MSCs is associated with its differentiation potential (Frykberg and Banks, 2015[10]). Therefore, the authors have been trying to differentiate MSCs towards target cell (e.g. keratinocyte, heart, hepatocytes etc.) (Al-Shaibani et al., 2016[2]; Elberry et al., 2016[8]).

MSC differentiation *in vitro* is typically performed by using “cocktails” that are composed of growth factors and signalling molecules (Sasaki et al., 2008[27]). Target host tissue-conditioned medium is one of the possible “cocktail” mixtures to differentiate MSCs into required functional cells. The conditioned medium contains various growth factors and cytokines that are released from cultured cells (Li and Fu, 2012[16]; Al-Shaibani et al., 2017[1]; Li et al., 2017[17]). Studies have shown that *in vitro* keratinocyte-conditioned medium (KCM) successfully promoted MSC differentiation towards keratinocyte like-cells (Sasaki et al., 2008[27]; Chavez-Munoz et al., 2013[3]). However, these differentiated cells lose undifferentiated status and regenerative potential of the stem cells. It is therefore assumed, that partial cell differentiation could help to maintain stem cell regenerative properties. Studies have revealed that partially differentiated MSCs (PMSCs) are even more effective than MSCs and improve bone healing (Peters et al., 2009[24]), liver (Elberry et al., 2016[8]) and cardiac function (Ling et al., 2011[20]). 

However, there is a lack of information on PMSC effectivity in a skin tissue regeneration and accumulation in the wounded area. Moreover, the most effective cell delivery (intradermal and intravenous) methods are not determined. In this study, we obtained PMSCs, evaluated alterations in their surface marker expression, regenerative potential and accumulation in the wounded area in a full-thickness mouse skin wound model *in vivo*. 

## Materials and Methods

### Experimental animals

C57BL/6J mice at the age of 6-8 weeks were bred and housed in our local breeding facility at the State Research Institute Centre for Innovative Medicine (Lithuania). All procedures were carried out in accordance with the institutional guidelines of the European Union and were approved by the Lithuanian Ethics Committee on the Use of the Laboratory Animals under the State Veterinary Service No. B1 - 866. Animals were maintained in the environment of controlled temperature (23 ± 1 °C). Food and water were provided *ad libitum. *

#### Isolation and cultivation of mouse compact bone-derived MSCs

Compact bone-derived mesenchymal stromal cells (MSCs) were isolated from C57BL/6J mice as previously described by Zhu et al. (2010[39]) with some modifications. Briefly, the bone cavities were washed until the bones became pale. They were cut into 1-3 mm^3^ chips and were transferred into the tissue culture flask and cultured in MSC growing medium, containing DMEM with high glucose (Life Technologies, USA), 5 % FBS (Lonza, Switzerland), 5 % pHPL (prepared according to Schallmoser and Strunk, 2009[28]) and 1 % of antibiotics (penicillin and streptomycin 10 000 U, Lonza, Switzerland)). Cultures were maintained at 37 °C in 5 % CO_2_. On the third day of cultivation, a medium was changed to remove non-adherent cells and tissue debris. Adherent cells were further cultured with a medium change in every 2-3 d. At approximately 80 % confluence, cells were harvested by treating with 0.25 % Trypsin/EDTA solution (Lonza, Switzerland) for 2 min at 37 °C. The cells of forth passage were used for further experiments. Tri-lineage *in vitro* differentiation potential was performed as previously described by Sasaki et al. (2008[27]). Each differentiation medium was changed every other day for 3 weeks. Osteogenic, adipogenic, chondrogenic differentiation potential was confirmed by staining with alizarin, oil red O and toluidine blue, respectively.

#### Isolation and cultivation of mouse DSKs

Primary mouse dorsal skin keratinocytes (DSKs) were obtained from the hairless C57BL/6J mouse newborns (2-4 days) according to Lichti et al. (2008[18]) with a slight modification. The skin was removed from the body. After processing and cleaning, the skin tissue was transferred to a 1 mg/ml dispase II solution (Merck Millipore, USA) and incubated at 4 °C overnight epidermal side up. Next day, epidermis layer was peeled off from dermis without applying excessive pressure. A single cell suspension was prepared by cutting epidermis and gently shaking with a 22G syringe. Cells were transferred through 70 μm nylon membrane, pelleted, washed twice with PBS and resuspended in keratinocyte growing medium composed of Dulbecco's Modified Eagle's Medium (DMEM) without calcium (Life Technologies, USA) supplemented with 5 % FBS, 5 % pHPL, 1 % antibiotics and 0.07 mM CaCl_2_ (Sigma, Germany). Cells were seeded in (previously prepared) rat-tail I collagen (Gibco, USA) coated tissue flasks and incubated at 37 °C in 5 % CO_2_. On the 4^th^-5^th^ day of culturing (at approximately 80 % confluence), cells were treated with 0.25 % trypsin/EDTA for 2 min at 37 °C. Detached cells (1×10^5^ cells/cm^2^) were replated in a Keratinocyte Serum Free Medium (KSFM) (Life Technologies, USA) with the supplements mentioned earlier.

#### Exposure of MSCs to KCM

Keratinocyte-conditioned medium (KCM) was used to differentiate MSCs towards keratinocyte-like cells as previously described by Chavez-Munoz et al. (2013[3]) with a slight modification. When DSKs reached 70 % confluence, medium was collected, centrifuged to remove any debris and diluted with fresh KSFM in equal parts (1:1). MSCs were exposed to freshly harvested and diluted medium - KCM every day for the following 14 days. 

#### Imaging flow cytometry 

Data acquisition was performed by using AMNIS FlowSight (EMD Millipore, USA). Cells were detached with 0.05 % Trypsin/ EDTA solution, washed twice with PBS, and incubated with antibodies according to the manufacturer's recommendations. At least 10 000 events (DSKs, MSCs and PMSCs) were acquired. Cell marker expression under 2 % was considered as negative.

DSKs, MSCs and PMSCs were identified for the expression of hematopoietic lineage (lin) markers using mixture of biotinylated antibodies (CD3e, CD11b, CD45R/B220, Ly-6G and Ly-6C (Gr-1), TER-119), CD34-PE (phycoerythrin) (all from BD Biosciences, USA); CD29-APC (alofikocyanin), Sca-1-PE, CD44-PE, streptavidin-PE (all from Miltenyi Biotec, Germany); CD117-APC, CD31-AF647 (Alexa Fluor), biotinylated cytokeratin18 (CK18) (all from Invitrogen, USA); CD71-FITC (fluorescein isothiocyanate), CD49f-PE, (all from Abcam, UK). Appropriate isotype match controls were used as negative. Data were analysed with AMNIS IDEAS software.

#### Cell sorting

CD71+ cells were sorted out by BD ARIA II (BD Bioscience, USA) from PMSC culture using CD71 antibody. The obtained culture was used for further experiments as separated PMSC_CD71+ cell subpopulation.

#### Cell proliferation assay

MSC proliferation was determined using the Cell Count Kit 8 (CCK-8) assay (Dojindo, Japan) according to the recommendation by the manufacturer. 2.5 × 10^3^ cells/well were seeded into a 96-well plate. The absorbance at 450 nm was determined by the multiplate reader Sunrise (Tecan, Austria). Cell proliferation was represented as a percentage and normalized to the control (DMEM). Three experiments were performed in triplicate.

#### The excisional dorsal full-thickness skin wound model

Wound model was accomplished according to Wong et al. (2011[36]) with a slight modification. C57BL/6J mice were individually anaesthetized using a subcutaneous injection of 0.5 % *bupivacaini hydrochloridum* (50 μl/mouse). The skin was shaved, cleaned and disinfected with 70 % ethanol. Excisional 6 mm full-thickness skin wound (including the *panniculus carnosus) *was created on the right side of the midline using sterile 6 mm round skin biopsy punch. The wound was left uncovered.

The animals with excisional full-thickness skin wounds were divided into 4 experimental cell groups (MSCs, PMSCs, DSKs and PMSC_CD71+ cells) and the control group (PBS). Each group consisted of 12 mice: 6 for *intradermal* and 6 for *intravenous* injections. Cell suspensions (1 × 10^6^ cells/mouse in 100 μl PBS) and PBS (100 μl/mouse) were injected intradermally around the wound at four injection sites or intravenously into the mouse tail vein.

Digital photographs were taken on the day of surgery (day 0) and on 1^st^, 2^nd^, 3^rd^, 4^th^, 7^th^, and 8^th^ day. Time of wound closure was defined as the time, at which wound bed was completely filled in with new tissue. Wound size was calculated using the formula D_n_ × 100 % divided by D_o_, where D_n_ is the area of the wound on the indicated day and D_o_ is the area of a full-thickness skin wound on the first surgery day. Wounds with a complete reepithelialization were considered as healed wounds. 

#### In vivo cell imaging 

MSCs, DSKs, PMSCs and PMSC_CD71+ cells were labelled with MitoTracker Deep Red (Life Technology, USA) and injected intradermally/intravenously into the experimental animals as described above. C57BL/6J mice were anaesthetized with isoflurane and transferred to the IVIS Spectrum Imaging System (PerkinElmer, USA). MitoTracker Deep Red labelled cells in the same animal were monitored on 1^st^, 2^nd^, 3^rd^, 4^th^, 7^th^ and 8^th ^day after transplantation. Each C57BL/6J mouse was imaged dorsally (laid on the abdomen). MitoTracker Deep Red labelled cell fluorescence signal was detected at wavelengths 680/20 nm with excitation at 640/30 nm. 

All captured images were analysed using Living Image software 4.1 (PerkinElmer, USA). To analyse the change of MitoTracker Deep Red fluorescence intensity, a 12 mm circle was set on the region of interest (ROI) around the injured area. ROI was selected based on the size of initial wound (6 mm), including the injection sites around the wound. The same ROI size was also placed on the control mice (wounded mice with an intradermal or intravenous injection of PBS) as the background reference. Fluorescence intensity of the ROI was presented as an average radiant efficiency normalized to the first wound measurement.

#### Histologic examination

Mice were sacrificed on the 8^th^ day after the last measurements. Skin samples including initial wound area and 2 mm of the surrounding skin were harvested using 8 mm biopsy punch. The wound areas and the surrounding healthy skin were fixed in 10 % neutral buffered formalin for 2 days. The samples were dehydrated in a series of ethanol solutions and then embedded in paraffin. From different tissue block levels, at least 5 sections, 4 μm thick were cut and stained with hematoxylin/eosin. The sections were examined under a light microscope.

### Statistical analysis

All *in vitro* and *in vivo *experiments were performed at least three times independently. Results were expressed as mean ± SD. Differences between groups were tested by Student's t-test. Results were considered statistically significant when p < 0.05.

## Results

### Characterization of the cells

**Figure 1:**
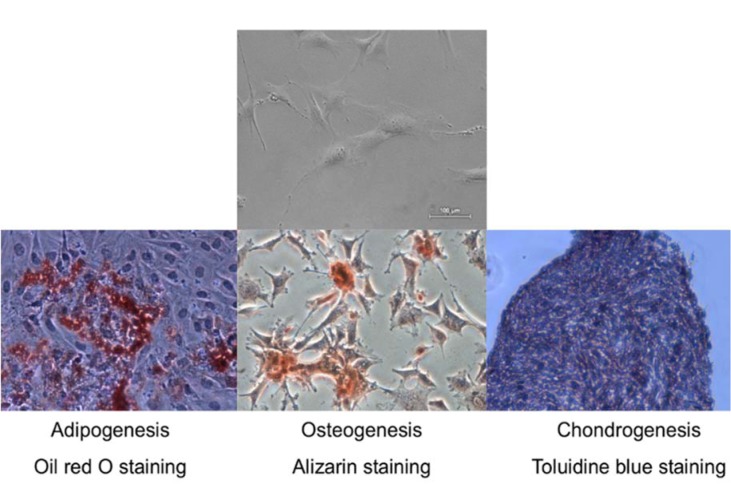
Tri-lineage differentiation potential of mouse compact bone derived mesenchymal stromal cells. Representative microscopic images of Oil red, Alizarin and Toluidine blue staining to confirm adipogenic, osteogenic and chondrogenic differentiation. Original magnification × 4

**Figure 2:**
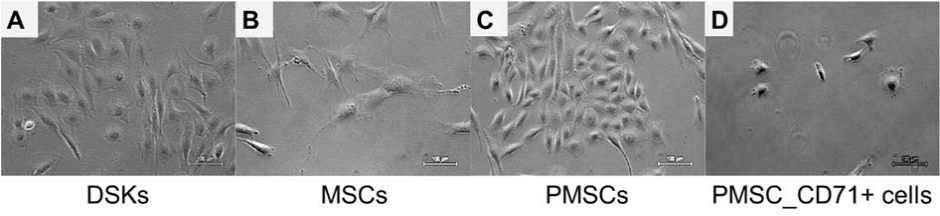
Morphology of the dorsal skin keratinocytes (DSKs), mesenchymal stromal cells (MSCs), partially differentiated MSC (PMSCs) and CD71 positive PMSC population (PMSC_CD71+). A. DSKs exhibited a typical cobblestone appearance. B. MSC typical spindle-shape form changed into a polygonal cobblestone-like appearance (C) after treatment with keratinocyte-conditioned medium for 14 days. D. PMSC_CD71+ cells shared keratinocyte-like appearance. Original magnification × 10

**Figure 3:**
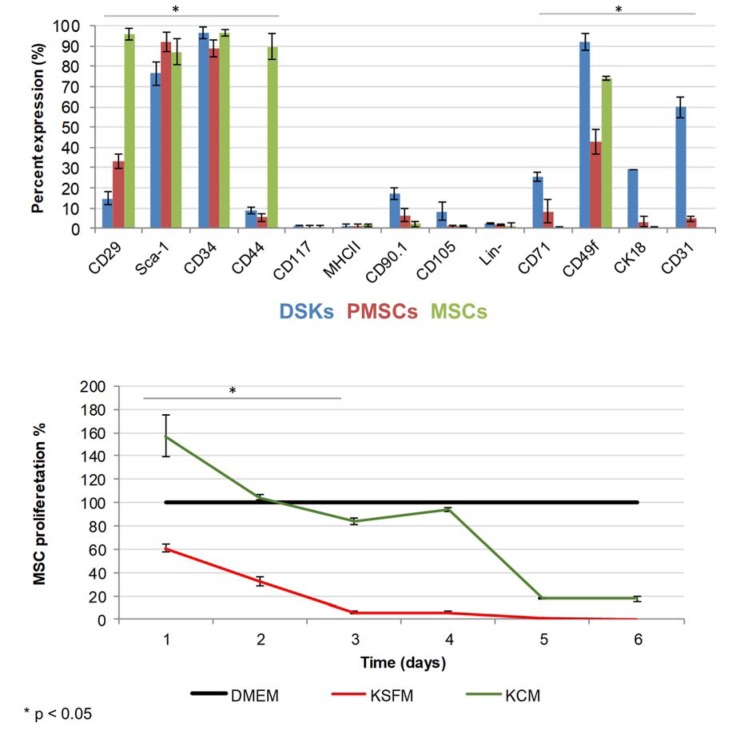
Figure 3: The expression of dorsal skin keratinocytes (DSKs), mesenchymal stromal cells (MSCs and partially differentiated MSCs (PMSCs) surface markers (above) and MSC proliferation changes (below). Cell proliferation presented as a percentage and normalized to the control (DMEM). KSFM - keratinocyte serum free medium, KCM - keratinocyte-conditioned medium. *p < 0.05

Compact bone-derived MSCs used in our experiments were established from compact bone explants of the C57BL/6J mice. Cultured cells were distinguished by fibroblastic shape morphology, typical MSC surface marker pattern and tri-lineage differentiation ability (Figure 1(Fig. 1), Figure 2 B(Fig. 2) and Figure 3(Fig. 3)). Phenotypic analysis of 4^th ^passage MSC cultures revealed that cells were positive for CD34 (97 ± 2 %), CD29 (96 ± 3 %), CD44 (90 ± 6 %), Sca-1 (87 ± 6 %), CD49f (74 ± 1 %) and negative for CD117, MHCII, CD105, CD71, CK18, CD31 and hematopoietic lineage markers (Figure 3(Fig. 3)). According to the surface marker description, CD34 is a negative marker for mouse MSCs, though other reports (Peister et al. 2004[23]; Sung et al. 2008[31]; Lin et al. 2012[19]), revealed that CD34 marker negative status in MSCs is just an artefact and it is associated with numerous variables, for example, mouse strain, tissue location or cell culture conditions. *In vitro* cell assays for osteogenic, chondrogenic and adipogenic differentiation showed positive approvals when stained with alizarin, toluidine blue and oil red O, respectively (Sasaki et al., 2008[27]) (Figure 1(Fig. 1)).

Primary mouse DSKs were obtained by enzymatic digestion of the epidermis layer. Cultured cells in early days were distinguished by typical keratinocytes polygonal cobblestone shape. Phenotypic analysis of primary DSKs were positive for CD34 (97± 3 %), CD49f (92 ± 4 %), Sca-1 (77 ± 6 %), CD31 (60 ± 5 %), CK18 (29 ± 3 %), CD71 (25 ± 2 %), CD90.1 (17 ± 3 %), CD29 (15 ± 3 %), CD44 (9 ± 2 %), CD105 (8 ± 4 %), and negative for CD117, MHCII and hematopoietic lineage markers (Figure 3(Fig. 3)). 

Keratinocyte differentiation potential has been defined by using CD49f and CD71 markers co-expression applied by other authors data (Tani et al., 2000[32]; Schlüter et al., 2011[29]). The population of quiescent keratinocyte stem cells (CD49f^+^/CD71^-^) comprised 53 ± 6 %, the population of cycling or transient amplifying keratinocytes (CD49f^+^CD71^+^) consisted of 35 ± 5 % and the population of early differentiating cells or differentiated cells (CD49f^-^CD71^-^) was of 11 ± 3 %.

### KCM effect on MSCs

KCM was used to differentiate MSCs to keratinocyte-like cells as previously described by Chavez-Munoz et al. (2013[3]). Within two weeks, approximately 70 % of MSCs changed their morphology from a spindle-shaped with 30 µm width and approximately 100 µm in length (Figure 2B(Fig. 2)) into a more similar to polygonal cobblestone shape with a width of approximately 40 µm (Figure 2C(Fig. 2)). The rest MSCs became shorter in length and more expanded in width. Observed DSKs in the same Figure (Figure 2A(Fig. 2)) exhibited cobblestone shape and were approximately 25 µm in diameter, very similar to the PMSCs (Figure 2C(Fig. 2)). PMSCs were positive for Sca-1 (92 ± 5 %), CD34 (89 ± 4 %), CD49f (43 ± 6 %), CD29 (33 ± 3 %), CD44 (5 ± 2 %), CD71 (8 ± 6 %), CD90.1 (7 ± 3 %), CD31 (5 ± 1 %), CK18 (3 ± 1 %) and negative for CD117, CD105, MHCII and hematopoietic lineage markers (Figure 3(Fig. 3)). 

CD71+ cells were sorted out from PMSC culture. The selected marker was atypical for MSCs and characteristic for DSKs. Isolated cells were used for further experiments as a separate PMSC_CD71+ cell subpopulation. 

The results of MSC proliferation showed the highest rate when cultured for 24 hours in the KCM. Results also demonstrated decreased proliferation in the KSFM (up to 39 %) and up to 56 % increase in KCM when compared with MSC proliferation in control culture medium (DMEM) (Figure 3(Fig. 3)). The results of MSC proliferation in KCM showed an accelerated growth over 90 % when compared to KSFM. However, this increased proliferation was short-term and started to decrease after 3 days of incubation with KCM. 

#### Wound healing analysis

**Figure 4:**
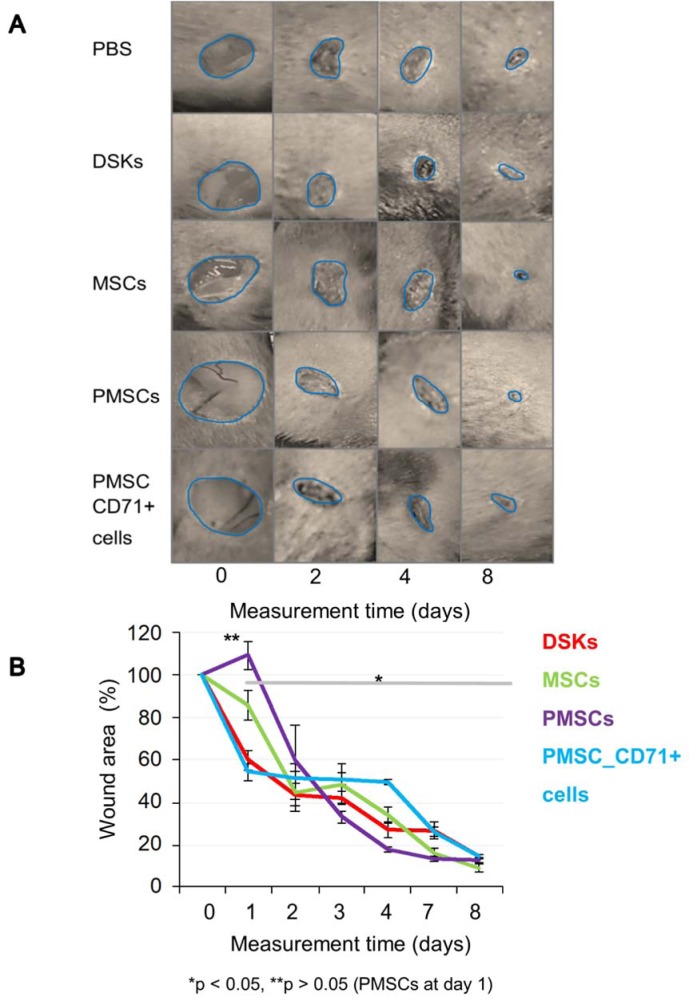
The influence of intradermally injected cells on wound contraction A. Images of mouse excisional wounds taken after surgery day (day 0) and on 2^nd^, 4^th^ and 8^th^ day post-wounding from a representative control (PBS), dorsal skin keratinocytes (DSKs), mesenchymal stromal cells (MSCs), partially differentiated MSCs (PMSCs) and CD71 positive PMSC population (PMSC_CD71+ cells) treatment B. Cell treatment effect on wound contraction. Error bars indicating standard deviation. n = 6 mice

**Figure 5:**
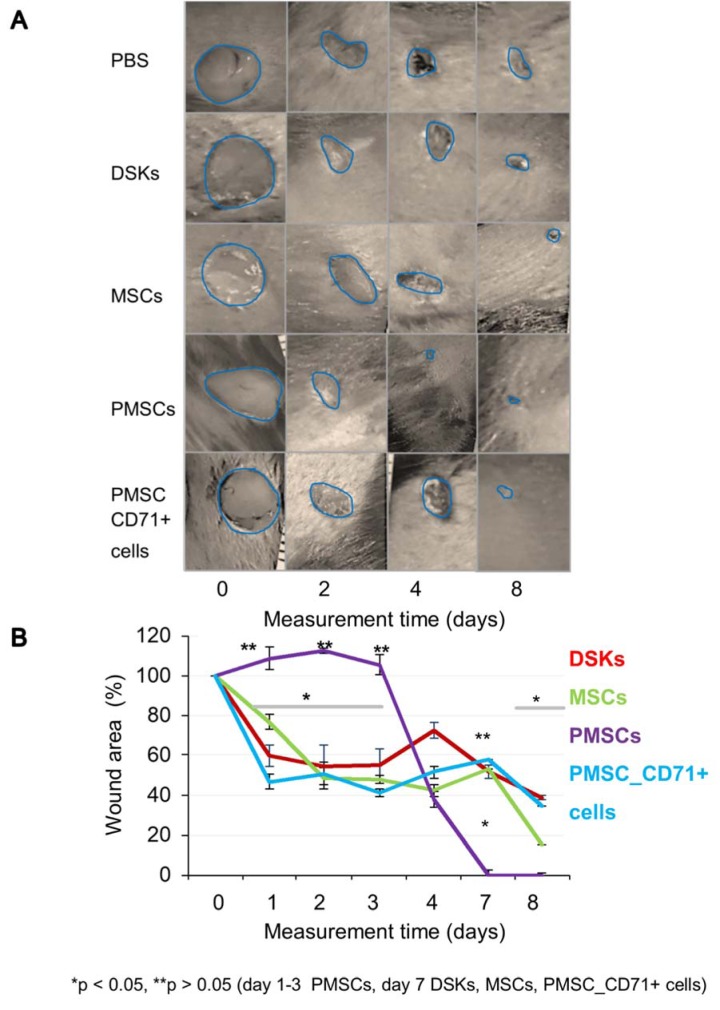
The influence of intravenously injected cells on wound contraction A. Images of mouse excisional wounds taken after surgery day (day 0) and on 2^nd^, 4^th^ and 8^th^ day post-wounding from a representative control (PBS), dorsal skin keratinocytes (DSKs), mesenchymal stromal cells (MSCs), partially differentiated MSCs (PMSCs) and CD71 positive PMSC population (PMSC_CD71+ cells) treatment B. Cell treatment effect on wound contraction. Error bars indicating standard deviation. n = 6 mice

The results showed that regardless of the cell delivery method (intradermal or intravenous), PMSCs, DSKs, MSCs PMSC_CD71+cells influence the process of mouse skin wound healing (Figure 4A and B(Fig. 4), Figure 5A and B(Fig. 5)). Enhanced wound healing effect was noticed from the first assessment day after cell injection. The wound healed by 90 % when treated intradermally with MSCs and PMSCs on the 8^th^ day, while the treatment with DSKs and PMSC_CD71+ cells wound healed 85 % (Figure 4A and B(Fig. 4)). The faster wound closure and completely healed wound on the 8^th^ day was observed when treated intravenously with PMSCs. Whereas treating with MSCs the wound was found healed 85 % and with DSKs and PMSC_CD71+ cells about 60 % (Figure 5A and B(Fig. 5)). Intradermally and intravenously injected group results showed that MSCs and PMSCs closed wounds faster in contradistinction to PMSC_CD71+ cells and DSKs.

### In vivo imaging 

**Figure 6:**
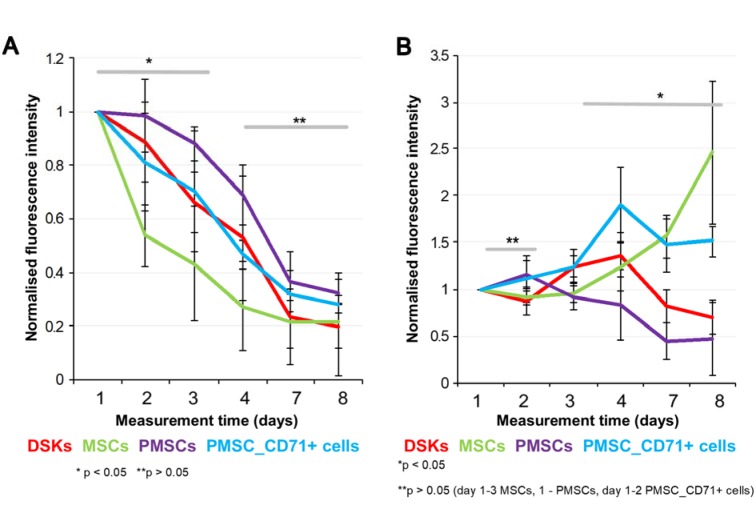
Cells fluorescence signal intensity in the full-thickness skin wound model. MitoTracker Deep Red labelled cells fluorescence intensities were estimated on 1^st^, 2^nd^, 3^rd^, 4^th^, 7^th^ and 8^th ^day after transplantation. A. Intradermally injected dorsal skin keratinocytes (DSKs), mesenchymal stromal cells (MSCs), partially differentiated MSCs (PMSCs) and CD71 positive PMSC population (PMSC_CD71+ cells relative amount in the region of interest (ROI) B. Intravenously injected DSKs, MSCs, PMSCs, PMSC_CD71+ cells in the ROI. Fluorescence intensity normalised to the first measurement. Error bars indicating standard deviation. n = 6 mice

**Figure 7:**
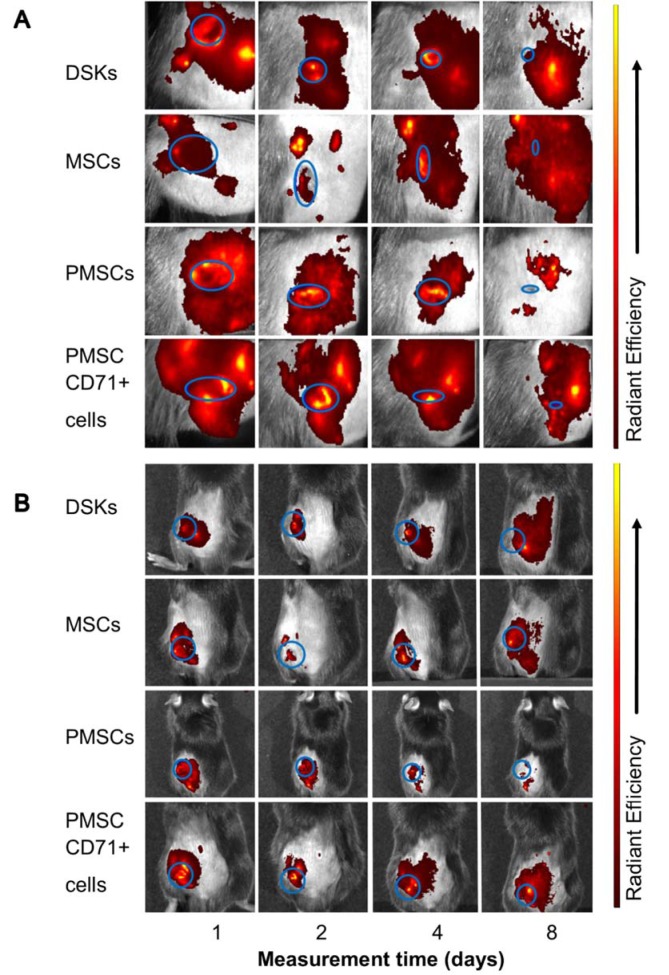
Visualisation of intradermally injected cells migration and accumulation in the C57BL/6J mouse. Images of MitoTracker Deep Red labelled cells in the same animal were taken on 1^st^, 2^nd^, 4^th ^and 8^th ^day after transplantation. A. The presence of dorsal skin keratinocytes (DSKs), mesenchymal stromal cells (MSCs), partially differentiated MSCs (PMSCs) and CD71 positive PMSC population (PMSC_CD71+ cells) in the wound area B. The presence of DSKs, MSCs, PMSCs, PMSC_CD71+ cells in the region of interest (ROI). Fluorescence intensity normalised to the first measurement. Error bars indicating standard deviation. n = 6 mice

**Figure 8:**
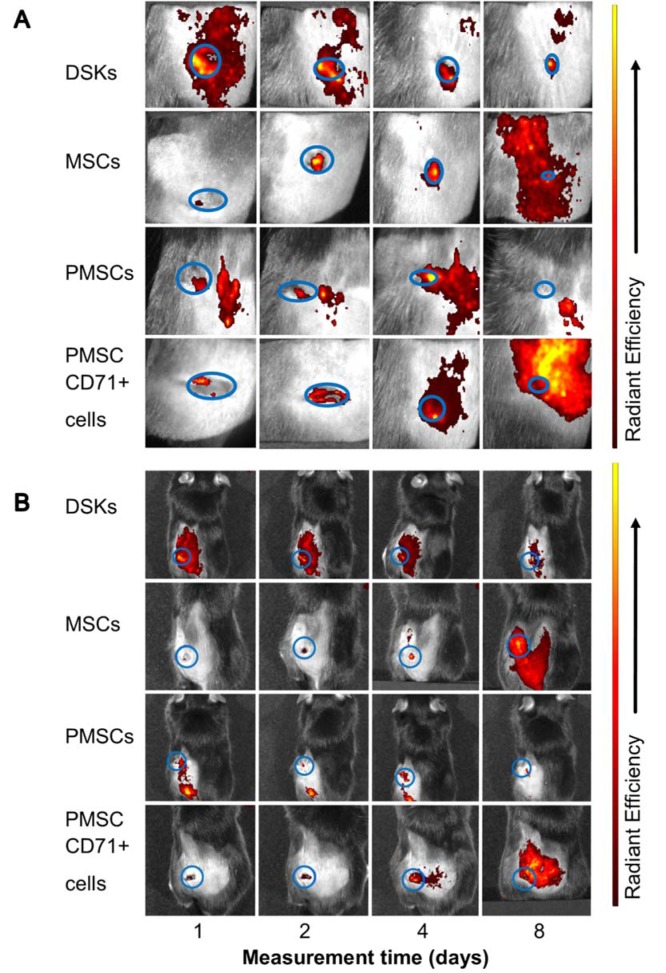
Visualisation of intravenously injected cells migration and accumulation in the C57BL/6J mouse. Images of MitoTracker Deep Red labelled cells in the same animal were taken on 1^st^, 2^nd^, 4^th ^and 8^th ^day after transplantation. A. The presence of dorsal skin keratinocytes (DSKs), mesenchymal stromal cells (MSCs), partially differentiated MSCs (PMSCs) and CD71 positive PMSC population (PMSC_CD71+ cells in the wound area B. The presence of DSKs, MSCs, PMSCs, PMSC_CD71+ cells in the region of interest (ROI). Fluorescence intensity normalised to the first measurement. Error bars indicating standard deviation. n = 6 mice

24 hours after wound surgery, MitoTracker Deep Red labelled injected cell fluorescence signal was measured in the ROI using IVIS Spectrum. Results showed that the intensity of ROI fluorescence varied among the cell groups (MSCs, PMSCs, DSKs and PMSC_CD71+ cells) due to different cell properties (cell size, staining efficiency and etc.) (Figure 6A and B(Fig. 6), Figure 7A and B(Fig. 7), Figure 8A and B(Fig. 8)). For this reason, fluorescence in the ROI was normalized to the first measurement in order to compare groups with each other. 

Intradermally injected PMSCs relative ROI fluorescence persisted most intensively and was up to 50 % more intense than with MSCs (Figure 6A(Fig. 6)). Meanwhile, in PMSC_CD71+ cells and DSK groups ROI fluorescence intensity was similar between the groups but higher than in MSC group. 

Fluorescent intensity in the ROI of all intravenously injected cells was increasing from the initial measurement point. In the PMSC group, labelled cells reached its fluorescence peak earlier than in other groups on 2^nd^ day, and then fluorescence intensity gradually decreased until the last measurement day. PMSC_CD71+ cells and DSKs reached theirs fluorescence maximum on 4^th^ day. The MSC fluorescence intensity in the ROI increased constantly until the last measurement day (Figure 6B(Fig. 6)).

### Histology

**Figure 9:**
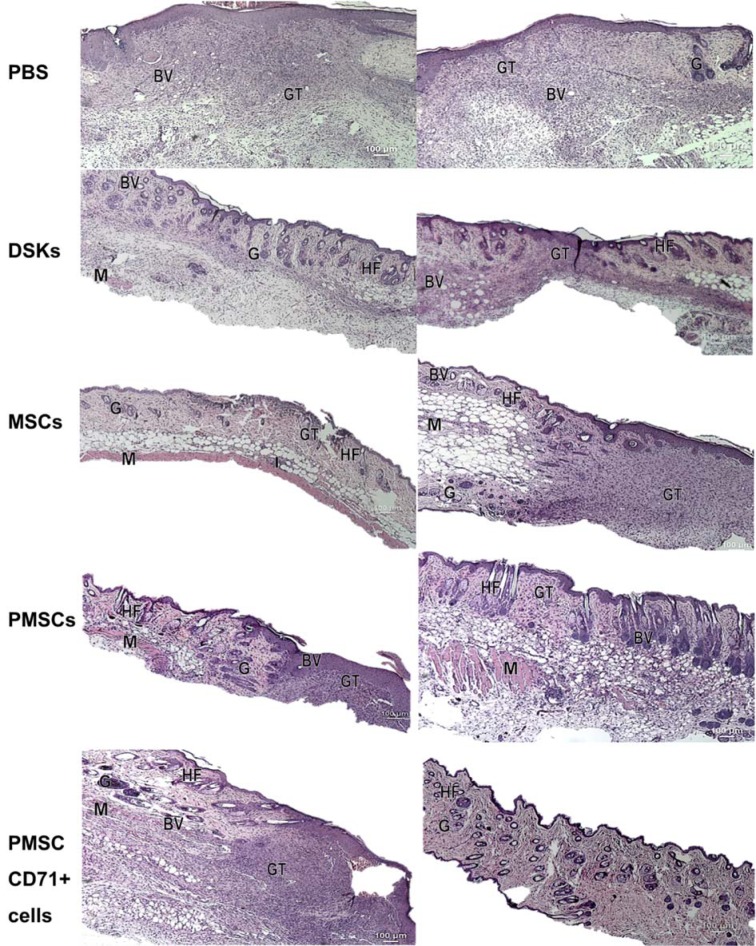
Representative images of hematoxylin and eosin stained mice skin wound section at day 8 treated with control (PBS), dorsal skin keratinocytes (DSKs), mesenchymal stromal cells (MSCs), partially differentiated MSCs (PMSCs) and CD71 positive PMSC population (PMSC_CD71+ cells). The left side represents the effect of intradermally injected cells and control on wound healing and the right side represents the effect of intravenously injected cells and control on skin wound healing. Abbreviation: GT granulation tissue, G: gland, BV: blood vessels, M: muscle, HF: hair follicle, I: cell infiltration. (Original magnification × 4)

Mouse skin biopsy specimens of intradermally or intravenously treated MSCs, PMSCs, DSKs and PMSC_CD71+ cells were examined using hematoxylin and eosin staining and are presented in Figure 9(Fig. 9). Histological examination of the control (PBS treated mice) specimens showed a successful reepithelialization, however, dermis elements failed to regenerate. The underlying wound site was filled with highly cellular granulation tissue containing numerous blood vessels, but hair and glands were not formed. 

Analysis of skin biopsy specimens of intradermally or intravenously treated MSCs, PMSCs, DSK and PMSC_71+ cells showed a successfully regenerated epidermis. The skin dermis site showed a mature granulation tissue; recover of adipose and muscular tissues with recuperated skin appendages. However, the differences in the amount of dermis elements were found among the cell groups. Skin biopsy specimens of intradermally treated MSC group had a persistence of inflammatory cell infiltration at the adipose tissue as well as a reduced amount of dermis elements in comparison with other cell treated animal groups. Skin biopsy specimens of MSCs intravenously treated mice had a granulation tissue residual. Skin biopsy specimens of intradermally treated PMSC group had a residual granulation tissue, while the mature and untreated skin view was detected in the intravenously treated cell group. However, the dermis elements in the DSK treated mice were still differentiating and no formed adipose tissue was found in both intradermal and intravenous treated animals. The dermis elements in PMSC_CD71+ cells were still differentiating in both intradermally and intravenously cell treated animals. In the intradermally PMSC_CD71+ cell treated animal group, there was a residual of granulation tissue, while in the intravenously treated group, there were no muscle or adipose tissues found.

## Discussion

Despite the rapid progress in the wound care, the majority of chronic wounds remain refractory to treatment. Inappropriate therapy leads to the wound infection that could cause high patient mortality. Even when wounds eventually respond to the treatment, the healing process usually leads to the fibrosis, scarring and thickening of the affected tissue, with the loss of functions. Cell-based therapy plays an important role in the field of the skin tissue regeneration, especially in the cases when applied treatments are unsuccessful. Studies show that MSCs possess a variety of useful properties for wound healing such as ability to migrate to the site of injury, participate in regeneration of damaged tissues, stimulate proliferation and differentiation, promote recovery of injured tissue through the growth factor secretion and matrix remodelling (Lee et al., 2016[14]; Oh et al., 2018[21]). 

However, the use of undifferentiated MSCs in clinic is negotiable due to the possible harm to patients. Moreover, there is evidence that partially differentiated MSCs are safe, well incorporated and even more effective than MSCs.

In this study, we aimed to modify MSCs with keratinocyte-conditioned medium (KCM) and to examine (undifferentiated) MSCs, partially differentiated MSCs (PMSCs) and differentiated cell (DSK and PMSC_CD71+ cell) accumulation in the wounded area as well as cell regenerative efficiency in a full-thickness skin wound model. Moreover, the influence of intradermal and intravenous cell delivery methods on wound healing was evaluated as well. 

The MSCs were isolated from C57BL/6J mice compact bone. The results showed that cells had a fibroblastic morphology (Figure 2B(Fig. 2)) and typical MSC surface markers pattern (positive for CD29, CD34, CD44, Sca-1, negative for MHCII, CD117, CD90, CD105 and hematopoietic lineage markers) and did not express dorsal skin keratinocyte cell (DSK) (CD31, CD71 and CK18) markers (Figure 3(Fig. 3)) (Peister et al., 2004[23]; Lee et al., 2008[15]; Lin et al. 2012[19]). The results of *in vitro *differentiation assay showed a positive staining for tri-lineage differentiation (Figure 1(Fig. 1)) (Sasaki et al., 2008[27]).

The DSKs were isolated from 2-3 days C57BL/6J mouse skin epidermis. Cells had cobblestone morphology (Figure 2C(Fig. 2)) and expressed keratinocyte cell surface markers: CD31, CD71, CD49f and CK18 (Figure 3(Fig. 3)). Co-expression of CD71 and CD49f results showed that the majority of our isolated primary DSKs were CD49f^+^/CD71^-^ and CD49f^+^CD71^+^. According to the literature, these cell populations are called quiescent keratinocyte stem cells and transient amplifying keratinocytes, respectively (Tani et al. 2000[32]; Schlüter et al. 2011[29]). These results revealed that the majority (88 %) of our isolated DSKs possessed a long-term proliferative capacity and the ability to secrete a variety of biological molecules such as growth factors and cytokines into the culture medium (Chavez-Munoz et al., 2013[3]). The results of MSC proliferation showed that KCM accelerated MSC activity. In order to maintain cells in an active proliferation phase, the culture medium had to be changed daily (Figure 3(Fig. 3)). 

There are several ways to promote cell differentiation. To differentiate MSCs towards the keratinocyte lineage we used KCM as previously described (Chavez-Munoz et al., 2013[3]; Kim et al., 2016[13]). After 14 days of MSC cultivation with KCM, the derived PMSC population was distinguished by altered morphology from MSCs (Figure 2(Fig. 2)). Phenotypic analysis of obtained population (PMSCs) showed the expression of epidermal lineage marker (CD31) and markers of keratinocytes (CD71, CK18). Moreover, the expression of MSC characteristic markers CD29 and CD44 was drastically decreased and acquired a similar level to the DSKs (Figure 3(Fig. 3)). Similar results were also obtained by other authors, where MSCs were modified by KCM and derived cells showed the presence of specific keratinocyte markers, such as cytokeratin-5, involucrin, filaggrin and stratifin (Chavez-Munoz et al., 2013[3]). According to our results and the data collected by Chavez-Munoz and colleagues, MSCs were differentiated only partly.

Most differentiated cell populations derived from PMSC culture were further purified. We used surface marker CD71 that is atypical for MSCs and characteristic for DSKs (Figure 3(Fig. 3)). Hereafter obtained population was considered as PMSC_CD71+ cells. It shared similar properties to DSKs. Cobblestone shape morphology (Figure 2A and D(Fig. 2)), regenerative potential (Figure 9(Fig. 9)), accumulation in the wounded area (Figure 6A(Fig. 6)) and migration ability to the wounded area were similar in both populations (Figure 6B(Fig. 6)). The following results suggest that KCM encouraged MSCs shift to keratinocyte cells. Obtained PMSCs share keratinocyte cell characteristics. 

The MSCs, PMSCs, DSKs and PMSC_CD71+ cell group regenerative efficacy was investigated in a full-thickness skin wound model. Cells, before injection, were labelled with MitoTracker Deep Red and monitored in the ROI.

All cell groups significantly accelerated wound healing process (Figure 4A and B(Fig. 4), Figure 5A and B(Fig. 5)). It has been previously demonstrated that MSCs increase the rate of wound closure in full thickness skin wounds (Ghosh et al., 2017[11]). Moreover, MSCs promote skin appendages regeneration (Xia et al., 2017[37]). In our study, MSCs and PMSCs reduced wound faster than DSKs and PMSC_CD71+ cells, also MSCs and PMSCs restored skin better than DSKs and PMSC_CD71+ cells (Figure 9(Fig. 9)). It is shown that MSCs promote wound healing through the increased reepithelization and granular tissue formation (Lee et al., 2016[14]; Uchiyama et al., 2017[33]). In our study, skin biopsy specimen granulation tissue was changed to collagen fibers in intradermally treated MSC group, while the residual in the intravenously treated MSC group skin granulation tissue still remained (Figure 9(Fig. 9)). Expressed collagen indicates the ability of MSCs to regulate extracellular matrix production (Lee et al., 2016[14]).

Recent data suggests that lower efficiency of MSC therapy is associated with impaired viability rate of injected cells (Lee et al., 2016[14]; Wang et al., 2016[35]) or MSC arrests in the lungs before reaching the target-injury site (Violatto et al., 2015[34]; Wang et al., 2016[35]; Oh et al., 2018[21]). However, our tested cell groups including MSCs successfully reached the target site and migration to the other organs was not detected (Figure 6A and B(Fig. 6), Figure 7A and B(Fig. 7), Figure 8A and B(Fig. 8)). Furthermore, our results showed that MSC presence in the wound was the lowest in comparison with other cells tested. Decrease of fluorescence intensity in the first measurement days in intradermally treated mice group was found out, while in intravenously treated group MSC amount was rising until the last day (Figure 6A and B(Fig. 6)). Our results suggest that rate of wound contraction is not directly related to the quantities of migrated MSCs in the wounded area (Figure 4B(Fig. 4) and Figure 5B(Fig. 5)). However, the persistence of MSCs in the wound resulted in better skin regeneration (Figure 9(Fig. 9)). Moreover, studies have shown that MSC-conditioned medium can modulate wound repair in the absence of MSCs (Li and Fu, 2012[16]; Al-Shaibani et al., 2017[1]; Li et al., 2017[17]). However, studies also demonstrated that MSC enhanced regeneration was more effective than MSC-conditioned medium alone (Sherman et al., 2017[30]). In our opinion, MSCs can participate in the skin wound healing and tissue regeneration indirectly through paracrine effect on cells (Lee et al., 2016[14]) and directly through cellular differentiation towards skin cells such as keratinocytes or pericytes (Isakson et al., 2015[12]; Rajabian et al., 2017[25]). The results of the PMSC relative amount in the wounded area showed most intensive signal from all cell groups (Figure 6A and B(Fig. 6) on 3^rd^ day). Although we found out that PMSC population was neither DSKs nor MSCs but it shared some common similarities of both. Wound contraction in the intradermally PMSC treated mouse group was as fast as in the MSCs (Figure 4B(Fig. 4)), and it was found to be even faster when mice were treated with PMSCs intravenously (Figure 5B(Fig. 5)). Moreover, histological analysis showed similar cell populations (MSC and PMSC) effect on tissue restoration (Figure 9(Fig. 9)). Both populations (PMSC and MSC) regenerated skin tissue appendages more effectively than DSKs and PMSC_CD71+ cells. However, MSCs restored skin better when it was injected intradermally into mice, while PMSCs - intravenously. Moreover, intravenously PMSC treated mice group, completely healed the wounds (Figure 9(Fig. 9)) and it even was similar to the undamaged skin. Furthermore, the nature of migration and the persistance of PMSC population in the wounded area was more similar to the differentiated cells (DSKs). It is important to highlight that the wound contraction (Figure 4B(Fig. 4), Figure 5B(Fig. 5)) and cell accumulation in the wound kinetics (Figure 6A and B(Fig. 6)) showed that PMSCs possess different properties than MSCs. These results suggest that wound contraction was not directly related to the quantity of migrated cells in the wounded area. In the PMSC treated mouse group, relative cell amount in the wound was decreasing during wound healing process, while in the MSC treated mice group, cell amount in the wound area was increasing. These results demonstrate that PMSCs retain stem cell regenerative potential and acquire keratinocyte cell morphology and surface marker expressions. Furthermore PMSCs migrate to the sites of tissue injury faster than other cell groups. 

In conclusion, our results showed that keratinocyte-conditioned medium encouraged MSC differentiation into keratinocyte cells. The obtained PMSC population maintained MSC regenerative potential and acquired keratinocyte cell characteristics that lead to increased migration and improved survival at the site of injury. It leads us to the conclusion that due to partial differentiation status, PMSCs can differentiate more rapidly into epidermal cell lineages thus causing faster and qualitatively improved wound healing than MSCs and DSKs. PMSCs could potentially be a novel alternative source of cells for skin tissue regeneration due to their therapeutic efficiency in healing wounds. Further studies are necessary to support these considerations.

## Formatting of funding sources

This research did not receive any specific grant from funding agencies in the public, commercial, or not-for-profit sectors.

## Disclosure of interest

All the authors declare that they have no conflict of interest.
